# Controversies in brachial plexus injuries

**DOI:** 10.1186/1753-6561-9-S3-A26

**Published:** 2015-05-19

**Authors:** Rodrigo Banegas

**Affiliations:** 1 Peripheral Nerve Institute, Denver Health Medical Center, Denver, 80204, Colorado, USA

## 

This presentation will summarize some of the controversies I found treating these devastating injuries.

The brachial plexus injuries are amongst the most challenging cases that a hand surgeon can face in his career. The normal variations of the brachial plexus anatomy, the complexity of the injured poli-trauma patients and the current standard algorithms of treatment can be questioned in light of the functional results.

Kerr has shown historically regarding the variation of the normal anatomy of the brachial plexus that must have implications on the clinical evaluation of the patient, electro diagnostics studies and the recovery from an injury.

Understanding the hand and the upper extremity as a sensory organ brings a debate about the current algorithm of treatment that prioritizes the motor recovery of elbow flexion, shoulder stability and external rotation over sensory recovery. The latter, unfortunately, has an absence of good treatment options.

The evaluation of the patient by MRI and electro-diagnostic studies is also controversial in the setting of high percentage of false positives and false negatives and the above mentioned anatomical variations.

Very long and risky microsurgery procedures have been postulated for motor recovery achieving a poor result in many cases. In my practice, a huge patient dissatisfaction after reconstruction of a complete brachial plexus injury is more a rule than an exception. The key factor on this dissatisfaction is the lack of sensation and in some of the cases the inability to regain voluntary control of free muscle transfers that have been connected to intercostal and/or phrenic nerve.

Another controversial issue is the presence of de-afferentiation pain. This pain is a central nervous pain that is caused by the avulsion of the rootless from the spinal cord. Unfortunately, no good options are available when this situation becomes chronic.

The cost and risk of reconstructing a complete brachial plexus injury cannot be afforded by some medical systems in light of the poor results.

I can divide the strategies to treat the complete brachial plexus injuries into the conventional or biological and into non-conventional or technological. In this presentation I will introduce a project I am working on its initial steps. The project is named under bridge, due to the fact that the idea behind is bridging the brachial plexus injury with technological resources. The theory behind is that the brain still has the memory of the affected upper extremity and that the brain activity can be transmitted to an exo-skeleton to provide a motor function. Then an artificial shield that scenes temperature, pressure and motion can transmit this to the sensory area of the brain. For all these, communicating interface are to be created to connect the brain with the exo-skeleton and the sensory shield.

